# Inhibitors of endocytosis prevent Wnt/Wingless signalling by reducing the level of basal β-catenin/Armadillo

**DOI:** 10.1242/jcs.155424

**Published:** 2014-11-15

**Authors:** Maria Gagliardi, Ana Hernandez, Ian J. McGough, Jean-Paul Vincent

**Affiliations:** 1 MRC's National Institute for Medical Research, The Ridgeway, Mill Hill, London NW71AA, UK; 2 Department of Systems Biology, Harvard Medical School, Boston, MA 02115, USA

**Keywords:** β-catenin, Wnt, Endocytosis, Signalling

## Abstract

A key step in the canonical Wnt signalling pathway is the inhibition of GSK3β, which results in the accumulation of nuclear β-catenin (also known as CTNNB1), and hence regulation of target genes. Evidence suggests that endocytosis is required for signalling, yet its role and the molecular understanding remains unclear. A recent and controversial model suggests that endocytosis contributes to Wnt signalling by causing the sequestration of the ligand–receptor complex, including LRP6 and GSK3 to multivesicular bodies (MVBs), thus preventing GSK3β from accessing β-catenin. Here, we use specific inhibitors (Dynasore and Dyngo-4a) to confirm the essential role of endocytosis in Wnt/Wingless signalling in human and *Drosophila* cells. However, we find no evidence that, in *Drosophila* cells or wing imaginal discs, LRP6/Arrow traffics to MVBs or that MVBs are required for Wnt/Wingless signalling. Moreover, we show that activation of signalling through chemical blockade of GSK3β is prevented by endocytosis inhibitors, suggesting that endocytosis impacts on Wnt/Wingless signalling downstream of the ligand–receptor complex. We propose that, through an unknown mechanism, endocytosis boosts the resting pool of β-catenin upon which GSK3β normally acts.

## INTRODUCTION

Extensive evidence suggests that signalling and endocytosis are intimately linked. Classically, endocytosis has been thought to downregulate signalling by reducing the number of accessible receptors, either through sequestration in endosomes or degradation in lysosomes. However, it is now clear that endocytosis can also contribute positively to signalling initiation or propagation. One obvious possibility is that active signalling complexes could persist on endosomes (Hupalowska and Miaczynska, 2012; Pálfy et al., 2012; Seto and Bellen, 2006). This would result in prolonging and, hence, boosting signalling. In addition, endocytosis could contribute to the core mechanism of signalling. For example, endocytosis could provide a platform (endosomes) where signalling components are activated. Specifically, it has been suggested that, because of their distinct lipid composition, early endosomes recruit Sara, a FYVE-domain protein that stimulates TGFβ signalling by facilitating the interaction between ligand receptors and the downstream effector Smad2 (Di Guglielmo et al., 2003; Hayes et al., 2002; Tsukazaki et al., 1998). Different mechanisms have been suggested for the role of endocytosis in Notch signalling (Le Borgne et al., 2005). For example, recent experimental evidence shows that endocytosis could mechanically induce a conformational change in the receptor, which in turn would contribute to its activation (Meloty-Kapella et al., 2012). Therefore, there are a variety of ways in which endocytosis could contribute to signalling (Constam, 2009).

Another signalling pathway that seemingly requires endocytosis is that mediated by β-catenin (also known as CTNNB1) in response to Wnt proteins. Wnt signalling, a pathway involved in multiple aspects of development and adult homeostasis (Clevers and Nusse, 2012), is initiated by the binding of a Wnt ligand to its cognate Frizzled receptor, a seven-pass transmembrane receptor, and the subsequent formation of a trimeric complex with LRP6/5 (a single pass co-receptor called Arrow in Drosophila). Formation of the trimeric complex leads to phosphorylation of the intracellular domain of LRP5 or LRP6 which then recruits Axin, along with APC and GSK3β, the core components of the so-called β-catenin destruction complex (Kim et al., 2013; Metcalfe and Bienz, 2011; Stamos and Weis, 2013). Such recruitment leads to inactivation of the destruction complex and, hence, the accumulation of β-catenin and the activation of target genes. Several mechanisms have been proposed for the inhibition of the destruction complex. According to a common view, the engagement of Wnt with its receptor would disrupt the scaffolding activity of Axin, thus preventing GSK3 from acting on β-catenin. Alternatively, it has been suggested that the presence of Wnt would prevent the release of phosphorylated β-catenin from the destruction complex to the proteasome, preventing newly synthesized β-catenin from being degraded (Li et al., 2012). Yet another model, referred to below as the sequestration hypothesis (Taelman et al., 2010; Vinyoles et al., 2014), is that Wnt triggers trafficking of Frizzled and LRP6, along with associated GSK3β, to the lumen of multivesicular bodies (MVBs). As a result, GSK3β would no longer have access to β-catenin and become unable to trigger its degradation. All models agree that Wnt signalling prevents GSK3 from acting on existing or newly synthesised β-catenin, consistent with the finding that genetic removal or inhibition of GSK3β triggers strong signalling activity (Coghlan et al., 2000; Siegfried et al., 1992). However, the models differ in the mode of GSK3β inhibition, which could occur by disruption, saturation or sequestration of the destruction complex. Although the sequestration hypothesis has not been extensively tested, it has the benefit of providing a simple explanation as to how endocytosis could contribute to signalling because endocytosis is a prerequisite to MVB targeting.

Evidence that endocytosis contributes to Wnt signalling comes from two main lines of investigation. In one, endocytosis was blocked by genetically interfering with Dynamin (van der Bliek and Meyerowitz, 1991) in *Drosophila* wing imaginal discs, whereas the other made use of crude chemical inhibitors of endocytosis in cell culture. In developing wing imaginal discs, Wingless signalling directs the expression of various target genes. One such gene, *senseless*, is expressed in the cells flanking the main source of Wingless, at the prospective dorso–ventral boundary (Jafar-Nejad et al., 2006). Another gene commonly used as a target is *distal-less*, which is expressed throughout most of the wing primordium (Neumann and Cohen, 1997; Zecca et al., 1996). Expression of both genes is reduced in cells expressing a dominant-negative form of Dynamin (Piddini and Vincent, 2009; Seto and Bellen, 2006). This is consistent with a role for Dynamin-dependent endocytosis in Wingless signalling. However, one could not exclude pleiotropic effects on cell viability and general transcription activity (Piddini and Vincent, 2009; Rives et al., 2006). Unfortunately, this possibility cannot be formally discounted because the expression of target genes does not provide a sufficiently fast measure of signalling activity. Measuring the level of nuclear β-catenin, which is called Armadillo in *Drosophila*, could allow a faster measure of signalling after induction. However, within the developing wing, changes in the level of Armadillo are not easily detected, presumably because of the large Wingless-insensitive pool associated with cell adhesion complexes. By contrast, in mouse L cells, which do not express cadherin, a marked increase in total β-catenin can be detected by western blot within 60 minutes of treatment with exogenous Wnt 3 (Blitzer and Nusse, 2006). In the presence of monodansylcadaverine, chlorpromazine or hypertonic sucrose, which are all believed to inhibit clathrin-mediated endocytosis (Heuser and Anderson, 1989; Phonphok and Rosenthal, 1991; Wang et al., 1993), exogenous Wnt no longer induces an increase in β-catenin levels (Blitzer and Nusse, 2006). Although this result suggests that clathrin-dependent endocytosis is required for Wnt signalling, separate investigations have suggested that caveolae-mediated endocytosis could also be important, perhaps by allowing LRP5 or LRP6 to accumulate in a lipid environment that promotes its phosphorylation (Yamamoto et al., 2006). In conclusion, despite numerous findings supporting a role for endocytosis in canonical Wnt signalling, contradictory data remain and a mechanistic understanding is still elusive (Gagliardi et al., 2008). Here, we first confirm with improved inhibitors of Dynamin that endocytosis is required for the response to Wingless in *Drosophila* cells. As we show, addition of these inhibitors leads to a marked decrease in β-catenin/Armadillo in both *Drosophila* and mammalian cells. These inhibitors also prevent the rise of β-catenin/Armadillo that is normally caused by blocking GSK3β. This latter finding, along with other data, suggest that signalling is unlikely to require internalisation of the Wnt ligand itself, or sequestration of GSK3β in MVBs. Instead, we suggest that endocytosis inhibitors negatively impact on β-catenin levels through a currently unidentified mechanism that does not involve modulation of GSK3β activity.

## RESULTS

### Wingless signalling requires Dynamin-dependent endocytosis

**Fig. 1.**
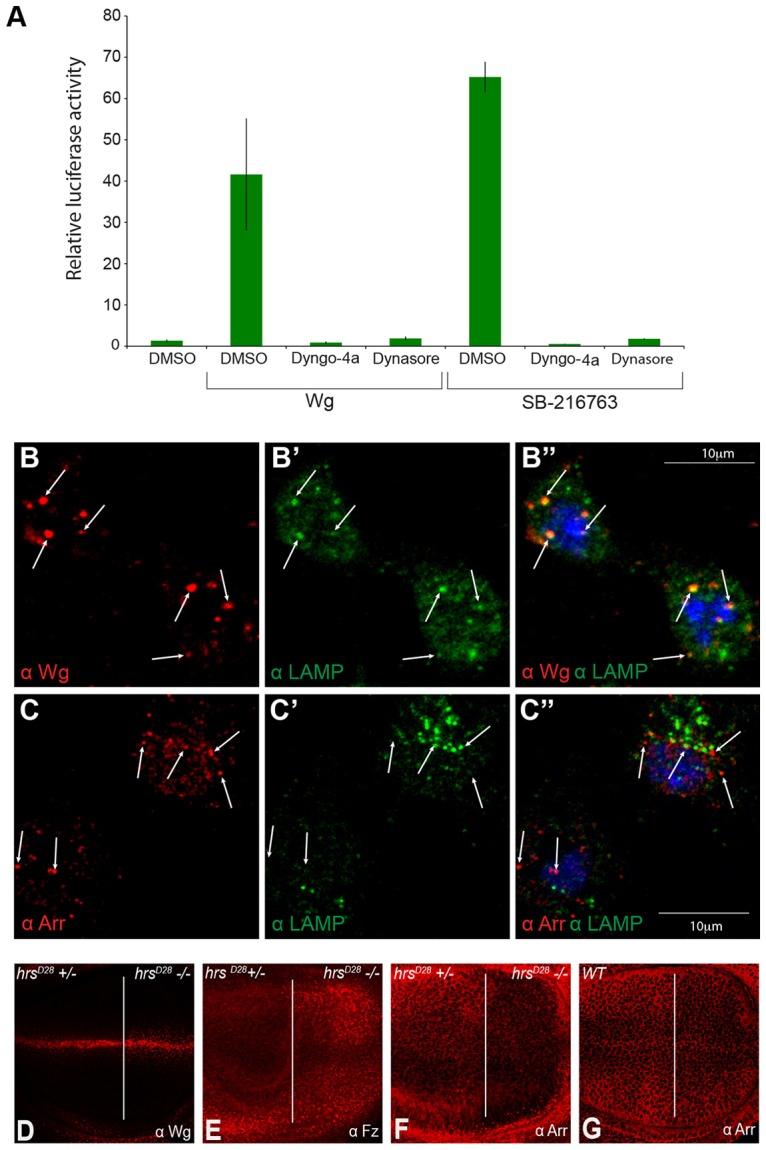
**Endocytosis is required for signalling, but internalised Arrow does not traffic to MVBs.** (A) Wingless signalling in S2R^+^ cells was activated either by Wingless-conditioned medium (Wg CM) or SB-216763 and the effect of endocytosis inhibitors on signalling activity was assessed, as indicated. Signalling activity was measured as the ratio of firefly to *Renilla* luciferase produced from the WISIR plasmid. The mean of triplicate measurements from a single trial±s.d. are shown. (B,C) Subcellular distribution of Wingless (Wg; B) and Arrow (Arr; C) relative to that of LAMP-1 in S2R^+^ cells 30 minutes after the addition of Wingless-conditioned medium. Although some Wingless colocalised with LAMP-1 (LAMP; white arrows in B), no substantial colocalisation between Arrow and LAMP-1 could be seen. (D,F) Loss of *hrs* (confirmed by the absence of β-galactosidase, not shown) was induced throughout the posterior compartment (right of the white line in D–G) with the Minute technique (Piddini and Vincent, 2009). This caused mild accumulation of Wingless and Frizzled2 (Fz) but not Arrow. (G) Wild-type (WT) imaginal disc stained with anti-Arrow.

We took advantage of two recently developed inhibitors of Dynamin, Dynasore and Dyngo-4a, to reassess the role of endocytosis in Wnt signalling (Macia et al., 2006; McCluskey et al., 2013). Signalling was assayed in *Drosophila* S2R^+^ cells (Yanagawa et al., 1998) that had been transfected with TOPFlash, a luciferase-based reporter of TCF and β-catenin activity (DasGupta et al., 2005; Gonsalves et al., 2011; Korinek et al., 1997). In accordance with previous reports (van Leeuwen et al., 1994), addition of Wingless-conditioned medium to S2R^+^ cells led to a ∼40-fold increase in luciferase activity (normalised against *Renilla* produced from a constitutive promoter). By contrast, Wingless conditioned medium did not elicit such a response in S2R^+^ cells that had been treated with Dyngo-4a or Dynasore (Fig. 1), confirming that Wingless signalling does require endocytosis. Importantly, both compounds also inhibited signalling activity triggered by an inhibitor of GSK3β, SB-216763. In control experiments, SB-216763 caused a strong increase in TOPFlash activity in S2R^+^ cells, whereas no or only a mild increase was seen upon concomitant treatment with Dyngo-4a or Dynasore (Fig. 1A). These results confirm previous evidence that intracellular transduction of canonical Wingless signalling requires endocytosis (Blitzer and Nusse, 2006; Seto and Bellen, 2006).

### Arrow/LRP6 does not traffic to MVBs in response to Wingless

One important component of the sequestration model is that Arrow (LRP5 or LRP6) should traffic to MVBs in response to Wingless because it is the intracellular domain of this receptor that has been proposed to carry GSK3β to the lumen of MVBs (Taelman et al., 2010). We first tested this prediction in S2R^+^ cells using LAMP1 as a marker for late endosomes and lysosomes. This is nominally a lysosomal protein but it is also present in MVBs (Rusten et al., 2007). No antibody marking solely and specifically MVBs could be found. Cells were treated with Wingless-conditioned medium for 2 hours and then fixed and stained with antibodies against Arrow, LAMP1 and Wingless. Fig. 1B shows that a substantial number of Wingless-containing punctae colocalise with LAMP1, in agreement with previous reports that Wingless is targeted to MVBs and lysosomes following engagement with its receptors (Piddini et al., 2005; Rives et al., 2006; Seto and Bellen, 2006). By contrast, Arrow did not colocalise with LAMP1 in Wingless-treated cells (Fig. 1C). This suggests that Arrow does not traffic to late endosomes with Wingless and that these proteins are likely to separate in an earlier compartment.

Next, we determined the impact of interfering with MVBs on the distribution of Wingless, Frizzled 2 (Fz2) and Arrow. To this end, we generated cells lacking Hrs, a protein that is required for normal MVB biogenesis (Lloyd et al., 2002). The posterior compartment of wing imaginal discs was made homozygous for a *hrs* mutant, hrs*D28*, using *hedgehog-gal4* and *UAS-flp* to trigger mitotic recombination in heterozygous animals. With this approach, combined with a cell lethal mutation on the homologous chromosome, most of the posterior compartment becomes reproducibly homozygous mutant (Piddini and Vincent, 2009). The resulting imaginal discs were stained with appropriate antibodies. As can be seen in Fig. 1D, the levels of Wingless and Fz2 increased in the mutant compartment (right-hand side of white line; see the figure legend and Materials and Methods) by comparison with those of the control anterior compartment. This was expected because both proteins are normally targeted to MVBs and lysosomes for degradation (Piddini et al., 2005). By contrast, loss of *hrs* caused a mild reduction, not an increase, in the levels of Arrow, confirming that Arrow probably follows a distinct trafficking route and is not normally targeted to MVBs for degradation (note that Rives et al., 2006 have previously reported trafficking of Arrow to MVBs, although this was based on tracking overexpressed tagged protein). Because of the lack of validated antibodies against *Drosophila* GSK3β, we could not determine whether GSK3β was trafficked to MVBs. Nevertheless, the evidence described above suggests that if it were, it could not be directed there by Arrow.

### Normal MVB biogenesis is not required for Wingless signalling

**Fig. 2.**
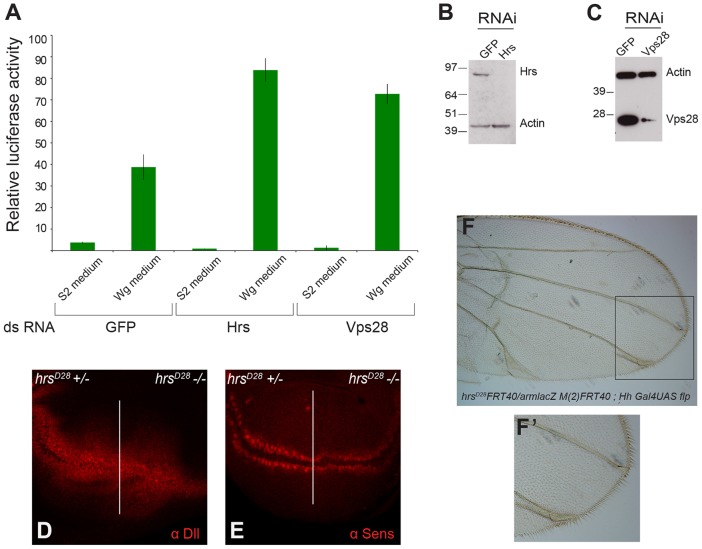
**Wingless signalling does not require functional MVBs.** (A) Knockdown (dsRNA) of *hrs* or *vps28* in S2R^+^ cells boosts the signalling that is induced by addition of Wingless-conditioned medium. Means±s.d. are shown. (B,C) Knockdown (RNAi) efficiency was confirmed by western blot analysis. (D,E) Expression of *distal-less* (*dll*) and *senseless* (*sens*) was largely unaffected by the loss of Hrs activity. The posterior compartment was rendered homozygous for an *hrs* mutant using the following genotype: *hrs^D28^ FRT40*/*arm-lacZ M*(*2*)*FRT40*; *hh-Gal4 UAS-Flp*. The slight reduction in *senseless* expression is probably due to developmental delay and had no impact on wing margin morphology, as indicated by panels F and F′ (F′ shows an enlarged image of the area highlighted in F), which show a wing of the same genotype. Note the normal bristles throughout the margin, including in the posterior compartment (lower half of the wing).

If sequestration of GSK3β does take place and is key to Wingless signalling, one would expect reduced MVB biogenesis to impair Wingless signalling. Therefore, we assessed the effect of reducing the activity of *hrs* or *vps28*, another gene involved in MVB biogenesis, on Wingless signalling in cultured cells and imaginal discs. First, S2R^+^ cells were treated with double stranded (ds)RNA against *hrs* or *vps28*, and the efficiency of knockdown was validated by western blotting (Fig. 2B,C). Wingless signalling was then assessed using the TOPFlash assay. As can be seen in Fig. 2A, Wingless-induced TOPFlash activity was not impaired by a strong reduction in either Hrs or Vps28. On the contrary, TOPFlash activity was mildly increased in RNA interference (RNAi)-treated cells (see also Rives et al., 2006). Next, the role of MVBs in Wingless signalling was assessed in imaginal discs by examining the pattern of expression of Senseless and Distal-less in *hrs* mutant cells. As shown above (Fig. 1D), posterior compartments that lacked *hrs* showed an accumulation of Fz2, consistent with an impairment of MVB function. In these imaginal discs, Distal-less continued to be expressed, seemingly normally. Senseless was also expressed in the normal pattern, albeit with a delay. Expression of Senseless is required for formation of the wing margin (Couso et al., 1994); therefore, as a further assay for Wingless signalling, we examined the margin of adult wings derived from mosaic larvae lacking *hrs* activity in the posterior compartment. Although wing veins were disrupted in the posterior compartment, possibly because another signalling pathway was affected, the posterior margin appeared normal (Fig. 2F). This confirmed that Wingless signalling is not substantially affected in *hrs* mutant tissue and that delayed Senseless expression is probably due to a general developmental delay caused by impaired trafficking.

### Internalisation of Wingless itself is not required for signalling

**Fig. 3.**
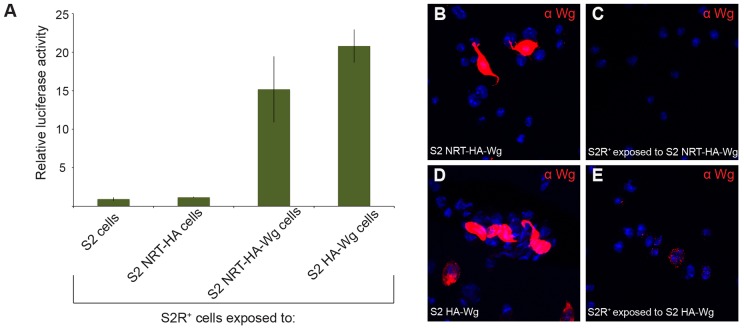
**Endocytosis, but not of Wingless, is required for Wingless signalling.** (A) S2R^+^ cells that had been transfected with the WISIR plasmid were co-cultured for 20 hours with S2 cells expressing NRT–HA, NRT–HA–Wingless or HA–Wingless, and luciferase activity was measured in the cell extracts. Relative luciferase activity is shown as the mean of triplicates from a single trial. Error bars represent the mean±s.d. (B–E) Internalisation assays with S2R^+^ cells co-cultured for 20 hours with S2 cells expressing either NRT–HA–Wingless or HA–Wingless (referred to as donor cells). Donor cells stained for Wingless are shown in B and D. These cells were washed off before staining the receiving S2R^+^ cells for Wingless. Internalised Wingless was detected in S2R^+^ cells that had been exposed to HA–Wingless-expressing cells (red dots, anti-Wingless in E) but not in S2R^+^ cells exposed to NRT–HA–Wingless-expressing cells (C). The endocytosed Wingless signal in S2R^+^ cells was distinctively weaker than that in transfected S2 cells, thus allowing us to distinguish the two cell types. Using this criterion, we infer that the Wingless-positive cells shown in E do not express Wingless. Wg, wingless.

Despite the evidence that endocytosis is required for Wingless signalling, the requirement for Wingless internalisation has not been formally considered. To address this question, we took advantage of a signalling-active fusion protein comprising Wingless and the type-2 transmembrane protein Neurotactin (NRT–Wingless) (Zecca et al., 1996). Because it is tethered to the membrane of the cells in which it is produced, NRT–Wingless is unlikely to be endocytosed by receiving cells. Indeed, recent evidence from mosaic imaginal discs suggests that NRT–Wingless does not transfer from one cell to another (Alexandre et al., 2014). We further tested this observation in cultured cells. S2 cells (Yanagawa et al., 1998) were used to express NRT–Wingless because they are non-adherent and can therefore be easily separated from S2R^+^ cells by gentle shaking. First, we confirmed that NRT–Wingless-expressing S2 cells can activate signalling in trans. ‘Responding’ S2R^+^ cells were transfected with the TOPFlash reporter and co-cultured for 20 hours with unresponsive S2 cells that expressed hemagglutinin (HA)-tagged proteins – NRT–HA (negative control), HA–Wingless (positive control) or NRT–HA–Wingless. As shown in Fig. 3A, NRT–HA–Wingless, as well as HA–Wingless triggered considerable TOPFlash activity in receiving non-expressing cells. The same co-cultures were used to measure internalisation. After 20 hours in co-culture, expressing S2 cells and receiving S2R^+^ cells were separated and stained with antibodies against HA. As expected, strong staining was seen in the cells that had been transfected with HA–Wingless or NRT–HA wingless (Fig. 3B,D). In responding cells that had been co-cultured with HA–Wingless-expressing cells, numerous HA-positive vesicles were detected (Fig. 3E), whereas no such vesicles were seen in cells that had been co-cultured with NRT–HA–Wingless (Fig. 3C). We conclude that, even though it is signalling competent, NRT–Wingless is not internalised in receiving cells. These findings, along with the evidence from mosaic imaginal discs (Alexandre et al., 2014), suggest that Wingless endocytosis is not required for signalling. This conclusion raises the question as to how inhibitors of endocytosis prevent Wingless signal transduction.

### Endocytosis inhibitors cause a rapid and reversible reduction of β-catenin/Armadillo levels

**Fig. 4.**
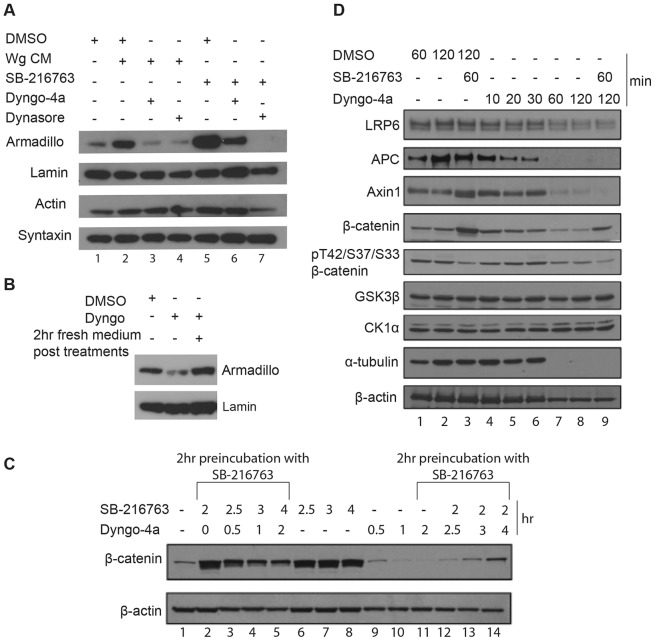
**Endocytosis inhibitors cause a decrease in the level of Armadillo/β-catenin both in stimulated and unstimulated cells.** (A) The level of Armadillo increased in S2R^+^ cells that had been treated with Wingless-conditioned medium (lane 2) or SB-216763 (lane 5). This was prevented by treatment with Dyngo-4a (lanes 3 and 6) or Dynasore (lanes 4 and 7). Lamin, Actin and Syntaxin levels were unaffected. (B) Dyngo-4a reversibly reduced the level of Armadillo in uninduced cells. (C) Dyngo-4a reduced signalling-induced accumulation of β-catenin in RKO cells. Cells were pre-incubated with SB-216763 to activate signalling and then exposed to a mixture of SB-216763 and Dyngo-4a. The total time of treatment with either drug is indicated. As in *Drosophila* cells, Dyngo-4a caused a decrease in β-catenin levels in unstimulated cells. A progressive decrease can be seen after 0.5, 1 and 2 hours of treatment with Dyngo-4a (no SB-216763) in lanes 9–11. (D) The effect of Dyngo-4a and SB-216763 on the level of various components of the Wnt pathway. LRP6, GSK3β and CK1α were largely unaffected, whereas the levels of APC and Axin1 dropped markedly 30–60 minutes after treatment with Dyngo-4a. SB-216763 caused an increase in the amount of β-catenin. This correlated with a decrease in phosphorylated β-catenin (pT42/S37/S33 β-catenin; lane 3), as expected because phosphorylated β-catenin reflects the activity of the degradation complex (Hernández et al., 2012). By contrast, the (mild) decrease in β-catenin caused by Dyngo-4a (lanes 7 and 8) is paralleled by a similar decrease in phosphorylated β-catenin, suggesting that Dyngo-4a impacts on the level of β-catenin through a mechanism that is independent of the destruction complex.

Wingless signalling is activated by preventing GSK3β from phosphorylating β-catenin. Indeed, chemical inhibitors of GSK3β trigger signalling, even in the absence of formation of a Wnt–receptor complex. For example, LiCl, which has been known for a while to block GSK3β activity (Stambolic et al., 1996), causes marked accumulation of β-catenin in L cells, and this is suppressed by concomitant treatment with monodansylcadaverine, chlorpromazine or hypertonic sucrose (Blitzer and Nusse, 2006). This provided an early indication that endocytosis could modulate signalling at or below the level of GSK3β, i.e. downstream of the ligand–receptor complex. This finding is at odds with the sequestration hypothesis; therefore, we sought to confirm it with more specific inhibitors. As an inhibitor of GSK3β, we chose SB-216763, which activates TOPFlash in the absence of exogenous Wnt in human cells (Coghlan et al., 2000). We found that SB-216763 activates TOPFlash in *Drosophila* S2R^+^ cells in a similar manner and therefore used this compound for subsequent investigation [the effect of other potent GSK3 inhibitors, such as CHIR98014 (Naujok et al., 2014; Ring et al., 2003) was not investigated]. We found earlier that TOPFlash activation with SB-216763 was prevented by concomitant treatment with Dynasore or Dyngo-4a (Fig. 1A). We conclude that inhibitors of endocytosis are unlikely to block Wingless signalling through preventing internalisation of the ligand receptor complex. Instead, it appears that endocytosis impacts on a more downstream event. The most immediate downstream sign of Wnt/Wingless signalling is the accumulation of β-catenin/Armadillo. We therefore investigated the effects of Dynasore or Dyngo-4a on the level of Armadillo in S2R^+^ cells. As shown in Fig. 4A, a 30 minute pre-treatment with either drug prevented the levels of Armadillo from rising in response to Wingless-conditioned medium or to SB-216763. In fact, it appeared that both drugs caused the level of Armadillo to dip below that seen in otherwise unstimulated cells, whereas the amounts of Actin and Syntaxin remained unchanged. This suggests that endocytosis could be required to maintain a sufficient steady-state level of Armadillo in resting cells. Indeed, addition of Dyngo-4a to S2R^+^ cells without any treatment to stimulate signalling led to a marked decrease in Armadillo (Fig. 4B). Interestingly, this effect was reversible – in cells that had been treated for 2 hours with Dyngo-4a and then allowed to recover in normal culture medium, Armadillo levels rose back to normal (Fig. 4B), suggesting that the effect of Dyngo-4a is transient and that cell viability was not adversely affected.

To confirm and extend the above findings, we next asked whether Dyngo-4a affected the level of β-catenin in mammalian cells in a similar manner. We chose human colon carcinoma RKO cells because they do not express E-cadherin and therefore lack the plasma-membrane-associated pool of β-catenin (Hernández et al., 2012). As a result, in these cells, a large fraction of β-catenin is subject to destruction complex regulation. In addition, RKO cells have intact Wnt components, unlike many colon cancer cells, which carry mutations in APC or β-catenin. Within 1 hour of treatment with Dyngo-4a, a reduction in the level of β-catenin was seen (compare lanes 10 and 1 in Fig. 4C). Likewise, Dyngo-4a reduced the rise in β-catenin caused by SB-216763 (compare lane 5 to lane 2 in Fig. 4C). We conclude therefore that inhibition of endocytosis leads to a reduction in the level of β-catenin, both in stimulated and unstimulated mammalian cells, as it does in *Drosophila* cells. Owing to the availability of suitable antibodies, it is possible to detect specifically the pool of human β-catenin that has been phosphorylated by GSK3β (at residues T41, S37 and S33; herein referred to as phosphorylated β-catenin). As shown recently (Hernández et al., 2012), this pool decreases upon the stimulation of signalling by exogenous Wnt or GSK3β inhibitors, and the level of phosphorylated β-catenin correlates inversely with the rate of β-catenin accumulation within the cell. We confirmed that the reduction in phosphorylated β-catenin caused by SB-216763 correlates with an increase in the level of total β-catenin (lanes 2 and 3 in Fig. 4D). However, the reduction of β-catenin caused by Dyngo-4 was not accompanied by increased levels of phosphorylated β-catenin (lanes 4–8 in Fig. 4D), indicating that endocytosis does not exert its effect on β-catenin levels by modulating the activity of the destruction complex. We next assessed the effect of Dyngo-4a on various components of the Wnt pathway. The levels of GSK3β and CK1α (also known as CSNK1A1) were unchanged, whereas those of APC and Axin1 dropped precipitously 30–60 minutes after treatment with the drug. A similar time course was seen for tubulin, although this could be specific to RKO cells because tubulin levels were unchanged in Dyngo-4a-treated *Drosophila* cells (not shown). The effect of Dyngo-4a on APC and Axin is remarkable because, so far, a reduction of either protein has been correlated with an increase in β-catenin levels, as expected because they form the scaffold of the destruction complex. By contrast, Dyngo-4a seems to trigger a concurrent decrease in Axin, APC and β-catenin.

## DISCUSSION

In this paper, we have used improved chemical inhibitors to confirm the requirement of endocytosis in canonical Wnt/Wingless signalling. Two indicators of signalling were used in *Drosophila* S2R^+^ cells: the stabilisation of Armadillo (*Drosophila* β-catenin) and the activation of a TOPFlash transcriptional reporter. Both were inhibited by either of two inhibitors of Dynamin, Dynasore and Dyngo-4a. These findings were extended by assessing the effect of Dyngo-4a on the level of β-catenin in human RKO cells that had been treated with a GSK3β inhibitor, SB-216763. Therefore, canonical Wnt signalling requires Dynamin-mediated endocytosis. Previous work has suggested that caveolin contributes to canonical Wnt signalling in vertebrate cells (Yamamoto et al., 2006). There is no Caveolin in *Drosophila*, and its role in caveolae could be fulfilled by Flotillins in this species. However, there is no evidence that Flotillins are required for Wingless signalling, even though they could contribute to the spread of Wingless within tissues (Katanaev et al., 2008). We suggest therefore that the requirement for Dynamin most probably reflects the involvement of Clathrin-mediated endocytosis, a suggestion that requires further investigation.

The requirement for endocytosis in Wnt signalling could be understood in the context of the sequestration model of Wnt signalling whereby Wnt triggers endocytosis of the receptor complex, along with the β-catenin destruction complex, and subsequent trafficking to the lumen of MVBs, thus preventing access of GSK3β to β-catenin. However, several observations are incompatible with this model. First, we find no evidence that, in *Drosophila* S2R^+^ cells, Arrow (*Drosophila* homologue of LRP5 or LRP6) traffics to MVBs in response to Wingless. Second, we show that impairing MVB biogenesis either in S2R^+^ cells or imaginal discs, does not adversely impact on signalling. Third, most tellingly, activation of signalling by a chemical inhibitor of GSK3β requires Dynamin-mediated endocytosis (see also Blitzer and Nusse, 2006). The latter finding strongly implies that endocytosis exerts its effect downstream of the Wnt–receptor complex, a suggestion that is difficult to reconcile with the sequestration hypothesis. To further dispel the assumption that endocytosis of Wnt/Wingless itself is required for signalling, we turned to a membrane-tethered form of Wingless and confirmed that it is not internalised by receiving cells and yet triggers downstream signalling.

As we found, inhibitors of endocytosis cause a sharp decrease in stimulated and unstimulated β-catenin/Armadillo levels both in *Drosophila* and human cells. This is probably due to increased degradation, although we cannot exclude an effect on biosynthesis. Two observations suggest that this decrease is not mediated by modulation of the destruction complex. First, in RKO cells, the drop in β-catenin did not correspond to an increase in phosphorylated β-catenin, which represents rapidly turning-over β-catenin (Hernández et al., 2012). Second, also in RKO cells, inhibition of endocytosis led to a marked decrease in the level of APC and Axin, two key components of the destruction complex. Therefore, in the presence of endocytosis inhibitors, inactivation of the destruction complex does not cause a substantial rise in β-catenin levels. Perhaps, in the absence of endocytosis, a complex of these three proteins becomes subject to an, as of yet, undefined degradation mechanism.

Irrespective of the mechanistic details, our results indicate the possible existence of an alternative, endocytosis-dependent, pathway that modulates the level of β-catenin/Armadillo. It is worth pointing out that other signalling pathways have been suggested to require endocytosis, although distinct mechanisms appear to be at work in each case. For example, endocytosis contributes to Hedgehog-mediated signalling by modulating the availability of Ptc at cilia (Yue et al., 2014) and to TGFβ signalling by providing a platform where activated receptors readily phosphorylate SMADs (Hayes et al., 2002).

One caveat of our work is that it relies on chemical inhibitors of endocytosis that have recently been found to have off-target effects (Park et al., 2013). An obvious alternative would be to use the temperature-sensitive mutation in Dynamin that is available in *Drosophila* (*shibire*[*ts*]; van der Bliek and Meyerowitz, 1991) in order to prevent endocytosis and assess the effect on Armadillo levels using western blots of imaginal discs. Unfortunately, in preliminary experiments, no notable change could be seen, most probably because in this tissue, much Armadillo is associated with adherens junctions and hence relatively stable. One way forward would be to introduce the *shibire*[*ts*] mutation in S2R^+^ cells through genome engineering (Bassett et al., 2013) and to then use such cells to further investigate the role of endocytosis in Wingless signalling and possibly screen for relevant components. Another issue concerns the number of proteins that are acutely affected by a reduction in endocytosis. The amount of several proteins were unchanged following the addition of Dyngo-4a to *Drosophila* and mammalian cells, an indication that the decrease in β-catenin/Armadillo induced by Dyngo-4a is not pleiotropic. However, it will be necessary to determine the proportion of the proteome that changes rapidly following inhibition of endocytosis.

## MATERIALS AND METHODS

### Cell culture

S2 and S2R^+^ cells, purchased from the *Drosophila* Genomic Research Center, were cultured in Schneider's medium containing 10% (v/v) fetal calf serum (FCS), 1% penicillin-streptomycin and 1% l-glutamine in an incubator at 25°C. RKO (human colon carcinoma) and L Wnt-3A (L-M[TK-] cells stably expressing mouse Wnt-3A, purchased from American Tissue Culture Collection; Manassas, VA), were cultured in Dulbecco's modified Eagle's medium (DMEM) containing 10% FCS and penicillin-streptomycin in a 37°C humidified incubator under 5% CO_2_.

Wingless-conditioned medium was prepared by incubating S2 pTub-Wg cells, kindly given to us by Roel Nusse (Stanford University, Stanford, CA) in standard Schneider's medium for 2 days. Wnt-3A-conditioned medium (Wnt-3A CM) and control medium were prepared according to the manufacturer's protocol. DNA transfection in S2 and S2R^+^ cells was performed using Effectene Transfection Reagent (Qiagen) following the procedure provided in the user manual.

### TOPFlash assay

Transcriptional activation through Wingless signalling was achieved using WISIR (Wingless Signalling Reporter), a plasmid designed and generated by Cyrille Alexandre (National Institute for Medical Research, London, UK). It contains the following elements: firefly luciferase under the control of eight consensus binding sites for TCF/LEF-1 (5′-AGATCAAAGGG-3′), a cassette comprising a histone H2A promoter driving histone H2A cDNA fused to yellow fluorescent protein (His2AYFP) and a copia promoter driving *Renilla* isolated from pCopia-Renilla. S2R^+^ cells were transfected with WISIR (0.4 µg) and the DNA or dsRNA of choice (0.2 µg). After 2 days, cells were treated with either 300 µl of Wingless-conditioned medium or S2 cell medium (control). One day later, cells were lysed in 1× Passive Lysis Buffer and both firefly and *Renilla* luciferase levels were assessed (Dual Luciferase Reporter Assay, Promega). Normalised data are expressed in relative luciferase units. They were averaged from triplicate assays. Error bars reflect s.d.

### Endocytosis inhibition

S2R^+^ or RKO cells were treated with Dynasore (200 µM, Sigma-Aldrich D7693), Dyngo-4a (100 µM, Abcam ab120689), or DMSO as a control, for 30 minutes before stimulation with Wingless- or Wnt-3A-conditioned medium for the indicated times. Cells were lysed with buffer as appropriate for western blotting or TOPFlash assay. To test the effect of blocking endocytosis on signalling activated by chemical inhibition of GSK3β, S2R^+^ cells were treated with Dynasore (200 µM) for 20 minutes followed by treatment with SB-216763 (20 µM, Sigma-Aldrich S3442) for up to 6 hours.

### Signalling and endocytosis assays using co-cultured cells

S2R^+^ cells were transfected with the WISIR vector (0.4 µg) and pUAST (0.6 µg). In parallel, S2 cells were transfected with either pMT-NRT-HA (0.5 µg), pMT-NRT-HA-Wingless (0.5 µg) or HA–Wingless (0.5 µg/µl) (Franch-Marro et al., 2008). The Metallothionein (MT) promoter was induced the next day by the addition of CuSO_4_ (0.7 mM). The S2R^+^ cells were then exposed to an equal number of S2 cells that had been transfected with the different constructs for 20 hours. S2 cells were washed off the S2R^+^ cells with PBS, which were then lysed with 1× Passive Lysis Buffer before luminescence readout. To assess endocytosis of NRT–HA–Wingless, NRT–HA (negative control) or HA–Wingless (positive control), S2 cells transfected with the relevant constructs were added in equal numbers to S2R^+^ cells and co-cultured for 20 hours. The S2R^+^ cells were then imaged after washing out the S2 cells with PBS.

### Immunoblotting

S2R^+^ cells were lysed using Triton Extraction Buffer (TEB) [50 mM Tris pH 7.5, 150 mM NaCl, 1 mM EDTA, 1% Triton X-100, protease inhibitor cocktail (Pierce), phosphatase inhibitor cocktail 2 (Sigma-Aldrich)]. The total protein concentration of the lysates was measured using the Bicinchoninic Acid assay (BCA) (Sigma-Aldrich, B9643) using bovine serum albumin (BSA) as a standard. Lysates were separated using SDS-PAGE (NuPAGE Tris-Acetate gels), and proteins were transferred to a PVDF membrane by using semi-dry electrophoretic transfer (14 V for 30 minutes). Membranes were blocked with 5% (w/v) Marvel milk dissolved in TBST (Tris-buffered saline with 0.1% Tween) for 1 hour at room temperature and then incubated with the appropriate primary antibody diluted in 5% Marvel milk overnight at 4°C. Membranes were then washed in PBS and incubated with a horseradish peroxidase (HRP)-conjugated secondary antibody diluted 1∶5000 in 5% Marvel milk for 2 hours at room temperature. Following extensive washing, enhanced chemiluminescence (ECL) (GE Healthcare) was used to detect the signals from the secondary HRP-conjugated antibody, and membranes were exposed to Amersham Hyperfilm ECL (GE Healthcare).

RKO cells were lysed with a buffer containing 50 mM Tris pH 7.6, 150 mM NaCl, 5 mM EDTA pH 8.0, 0.5% NP-40, protease inhibitor cocktail (Roche), 1 mM phenylmethylsulfonyl fluoride and the phosphatase inhibitors sodium fluoride (10 mM), p-nitrophenylphosphate (20 mM), β-glycerophosphate (20 mM), sodium orthovanadate (1 mM), okadaic acid (1 mM) and mycrocystin-LR (1 mM). The total protein concentration of the lysates was measured using the Bradford method (BioRad, Hercules, CA), using BSA (Sigma-Aldrich, St Louis, MO) as a standard. Proteins were resolved by using linear SDS-PAGE (7.5% acrylamide) and transferred to nitrocellulose membranes using electrophoretic transfer. The nitrocellulose membranes were blocked with blocking buffer (TBS, 0.1% Tween-20, 5% non-fat dry milk) for 0.5 hours at room temperature. The membranes were incubated overnight at 4°C with a primary antibody, which was diluted 1∶500 to 1∶2000. After extensive washing with TBS with 0.1% Tween-20, the membranes were incubated for 30 minutes at room temperature with a HRP-conjugated secondary antibody (Jackson ImmunoResearch, West Grove, PA), which was diluted 1∶10,000. As described above, enhanced chemiluminescence was used to detect HRP signals, and membranes were exposed to Amersham Hyperfilm ECL.

Antibodies against the following proteins were used: Armadillo (N2 7A1, DSHB; 1/5000), Lamin (ADL84.12, DSHB; 1/10,000), Actin (224-236-1, DSHB; 1/5000), Syntaxin (8C3, DSHB; 1/1000), Hrs (Hugo Bellen 1/20,000), β-catenin (BD Transduction Laboratories), phosphorylated β-catenin (at residues T41, S37, S33) (Cell Signalling), Axin1 (AF3287, R&D Systems), β-actin (Sigma) and α-tubulin (NeoMarkers).

### Immunostaining

Wing imaginal discs were fixed in 4% paraformaldehyde (PFA) in PBS at room temperature for 20 minutes and extensively washed in PBS. Fixed discs were permeabilised in 0.1% PBT (0.2% BSA and 0.1% Triton X-100 in PBS) and then blocked for 1 hour at room temperature in 4% FCS in PBT. Discs were incubated with primary antibodies diluted in blocking buffer overnight at 4°C and then at room temperature for 2 hours in Alexa-488- or -555-coupled secondary antibody diluted in PBT. After incubation, discs were again washed in PBT and mounted on slides in Vectashield.

Cultured cells were washed in PBS and then fixed in 4% PFA for 10 minutes. After another wash in PBS, they were quenched with 50 mM NH_4_Cl in PBS for 10 minutes, permeabilised for 10 minutes with 0.1% Triton X-100, blocked for 30 minutes with blocking solution (2% FCS and 2% BSA in PBS) and subsequently incubated in the appropriate primary antibody diluted in 50% blocking solution in PBS for 1 hour. After several washes in PBS, cells were incubated in Alexa-conjugated secondary antibody, also diluted in 50% blocking solution in PBS for 30 minutes. Cell preparations were mounted onto slides using MOWIOL 4-88 reagent (Calbiochem) reconstituted following the protocol provided. Images of stained imaginal discs and cells were obtained using a laser Leica SP5 scanning confocal microscope and analysed using Fiji software.

Antibodies used for immunostaining were: guinea pig anti-Senseless (Hugo Bellen, Baylor College of Medicine, Houston, TX; 1/500), guinea pig anti-Arrow (Suzanne Eaton, MPI Dresden, Germany; 1/500), rabbit anti-Frizzled2 (against amino acids 232–251; 1/100), mouse anti-Wingless (DSHB, 4D9; 1/500), anti-Distal-less (Ian Duncan, Washington University, St Louis, MO; 1/800), rabbit anti-LAMP 1/300 (Abcam 24170).

### Flies

To generate imaginal discs with *hrs* mutant posterior compartments, *hrs^D28^ FRT40/CTG* (*CyO*, *Twist-GFP*) flies were crossed with flies of genotype arm-lacZ M(2) FRT40/GlBc; hh-Gal4 UAS-Flp/+ to generate larvae of genotype *hrs^D28^ FRT40/arm-lacZ M*(*2*)*FRT40*; *hh-Gal4 UAS-Flp.* To assess the phenotypes of adult wings, wings of the desired genotype were dissected in isopropanol and mounted in Euparal (Agar Scientific) followed by incubation overnight at 65°C. Wings were analysed and photographed using Zeiss Axiophot 2.
